# Assessment of an integrated knowledge translation intervention to improve nutrition intakes among patients undergoing elective bowel surgery: a mixed-method process evaluation

**DOI:** 10.1186/s12913-021-06493-2

**Published:** 2021-05-27

**Authors:** Megan Rattray, Andrea P. Marshall, Ben Desbrow, Michael von Papen, Shelley Roberts

**Affiliations:** 1 School of Allied Health Sciences, Griffith University, Gold Coast Campus, Gold Coast, QLD 4222 Australia; 2 School of Nursing and Midwifery, Griffith University, Gold Coast Campus, Gold Coast, QLD 4222 Australia; 3 Menzies Health Institute Queensland, Griffith University, Gold Coast, Australia; 4 Gold Coast Hospital and Health Service, 1 Hospital Boulevard Southport Qld, Gold Coast, 4215 Australia

**Keywords:** Integrated knowledge translation, Early oral feeding, Knowledge translation, Process evaluation, Clinical nutrition

## Abstract

**Background:**

A large evidence-practice gap exists regarding provision of nutrition to patients following surgery. The aim of this study was to evaluate the processes supporting the implementation of an intervention designed to improve the timing and adequacy of nutrition following bowel surgery.

**Methods:**

A mixed-method pilot study, using an integrated knowledge translation (iKT) approach, was undertaken at a tertiary teaching hospital in Australia. A tailored, multifaceted intervention including ten strategies targeted at staff or patients were co-developed with knowledge users at the hospital and implemented in practice. Process evaluation outcomes included reach, intervention delivery and staffs’ responses to the intervention. Quantitative data, including patient demographics and surgical characteristics, intervention reach, and intervention delivery were collected via chart review and direct observation. Qualitative data (responses to the intervention) were sequentially collected from staff during one-on-one, semi-structured interviews. Quantitative data were summarized using median (IQR), mean (SD) or frequency(%), while qualitative data were analysed using content analysis.

**Results:**

The intervention reached 34 patients. Eighty-four percent of nursing staff received an awareness and education session, while 0% of medical staff received a formal orientation or awareness and education session, despite the original intention to deliver these sessions. Several strategies targeted at patients had high fidelity, including delivery of nutrition education (92%); and prescription of oral nutrition supplements (100%) and free fluids immediately post-surgery (79%). Prescription of a high energy high protein diet on postoperative day one (0%) and oral nutrition supplements on postoperative day zero (62%); and delivery of preoperative nutrition handout (74%) and meal ordering education (50%) were not as well implemented. Interview data indicated that staff regard nutrition-related messages as important, however, their acceptance, awareness and perceptions of the intervention were mixed.

**Conclusions:**

Approximately half the patient-related strategies were implemented well, which is likely attributed to the medical and nursing staff involved in intervention design championing these strategies. However, some strategies had low delivery, which was likely due to the varied awareness and acceptance of the intervention among staff on the ward. These findings suggest the importance of having buy-in from all staff when using an iKT approach to design and implement interventions.

**Supplementary Information:**

The online version contains supplementary material available at 10.1186/s12913-021-06493-2.

## Contributions to the literature

- Efforts to close the gap between evidence-based recommendations and current practice for the provision of timely and adequate nutrition to patients following elective bowel surgery are warranted to improve patient and healthcare outcomes. This study describes the co-development of a complex nutrition intervention with knowledge users and presents an evaluation of the processes supporting implementation in practice.
- Certain strategies were well adopted by staff, while others were not, which appeared to be due to variability in the delivery of awareness and education sessions to staff and their direct involvement in co-designing the intervention.
- Our findings suggest future work intending to implement interventions and guidelines should place high importance on knowledge user engagement in order to optimize intervention success.

## Background

Evidence-based guidelines (EBGs) for the prescription of nutrition to non-critically ill patients undergoing bowel surgery [1–3] reinforce the benefits and safety of reintroducing liquids and solids within 24 h after surgery to improve patient and hospital outcomes [4–8]. Further, patients who receive timely and adequate nutrition following surgery are more likely to meet their energy and protein needs while in hospital, reducing the risk of protein-energy malnutrition (PEM) and its associated consequences [9–13]. However, despite clear EBGs, several studies describing nutrition care practices and intakes among patients who have undergone colorectal (i.e. bowel) surgery have consistently demonstrated variable and often poor adherence to EBG recommendations [14–17]. Collectively, professional, patient and organisational factors have been reported to contribute to suboptimal feeding practices and poor nutritional intakes among surgical patients [16, 18–20]. Hence, efforts to close the gap between EBG recommendations and current practice for the provision of timely and adequate nutrition to patients following colorectal surgery are warranted.

Knowledge translation (KT) has emerged as an effective method for moving research findings from academic journals into use across a range of healthcare settings [21]. Many models and frameworks for supporting the translation of knowledge into practice have been developed, outlining the steps necessary to design, implement and evaluate evidence-based interventions [22]. The Knowledge to Action (KTA) framework proposed by Graham et al. [23] is one such model, consisting of seven cyclic steps, referred to as the Action Cycle. Importantly, collaboration between knowledge creators (i.e. the research team) and knowledge users (i.e. patients, family members and healthcare professionals (HCPs)) at each step is paramount to achieve relevant, applicable and impactful results [21]. Beneficial outcomes have been widely reported with the use of this approach [24]. This partnership is referred to as integrated knowledge translation (iKT), defined as a collaborative approach to research, whereby knowledge creators and knowledge users synergistically work towards translating evidence into practice to optimise health care [25].

Our team used an iKT approach, guided by the Action Cycle of the KTA framework to develop, implement and evaluate an intervention designed to improve nutrition care practices and dietary intakes among patients who undergo colorectal surgery. Considering KT interventions typically demand behaviour change in complex environments [26, 27], an evaluation of the processes supporting implementation is essential to understand why an intervention did or did not achieve its intended aims, and how implementation can be optimised in the future [26, 28]. As such, the primary aim of this study was to evaluate the processes supporting the implementation of a complex nutrition intervention in an inpatient surgical setting. This practical example will provide useful information for clinicians seeking to translate nutrition guidelines into practice in their own settings.

## Methods

### Study overview

**Fig. 1:**
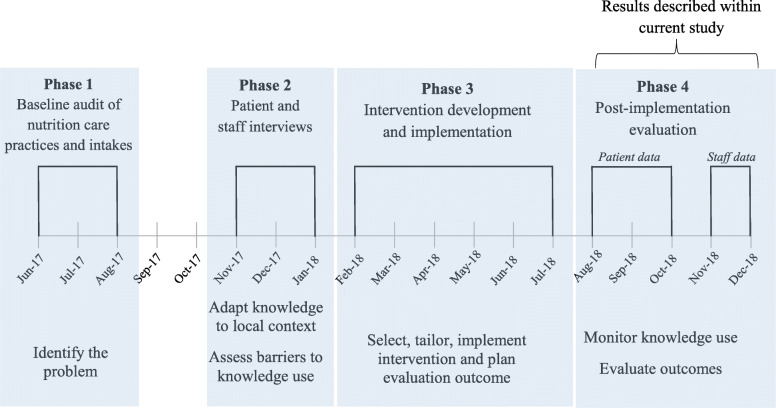
Project timeline aligned with Action Cycle of Knowledge to Action framework

This study is part of a larger, four-phased program of research, employing an iKT approach [29] guided by the Action Cycle of the KTA framework [23] to design, implement and evaluate an intervention designed to improve the timing and adequacy of nutritional intakes among patients who undergo bowel surgery (Fig. 1). This paper describes the process evaluation (Phase 4) of the intervention. The relevant hospital and university Human Research Ethics Committees approved all study procedures (reference numbers: HREC/17/QGC/101 and GUREF/2017/389). The reporting of this study was guided by The Template for Intervention Description and Replication (TIDieR) checklist [30] and the Consolidated Criteria for Reporting Qualitative Research [31] (Supplementary Material 1 and Supplementary Material 2, respectively).

### Study design and setting

A prospective, mixed-methods design, underpinned by Moore’s process evaluation framework [32] was utilised. Process evaluation domains assessed included intervention reach and delivery and participant responses to the intervention. These were evaluated by analysing quantitative and qualitative data collected from patients and HCPs. The study was conducted in one GI surgical ward at a large (750 bed) tertiary teaching hospital located in Queensland, Australia, where approximately 200 elective small and large bowel resections are performed each year. The colorectal surgical team consisted of six consultants, two fellows, two registrars, one resident and two interns. Within this setting, Enhanced Recovery After Surgery (ERAS) guidelines were not formally in use; feeding and other postsurgical orders were prescribed at the discretion of each surgeon. An electronic foodservice system (Delegate Software, Australia) was in operation at the hospital, whereby nursing staff entered patients’ dietary prescriptions into the system and patients ordered their main meals via a bedside patient entertainment system screen (≥3 h in advance of meal delivery). Patients were delivered a generic meal for the diet they had been allocated if they did not place a meal order.

### Intervention development

In line with an iKT approach, the intervention was co-developed by the research team and knowledge users. The research team engaged knowledge users in two ways: 1) establishing and consulting with a *Nutrition Reference Committee,* which included patients and HCPs from multiple disciplines, developed specifically for the project; and 2) engaging in regular group and one-on-one discussions with staff on the study ward who were not part of the committee (Supplementary Material 3). Considering the provision of postoperative nutrition care required a multidisciplinary approach, *Nutrition Reference Committee* members included medical staff (surgeons), dietitians, nurses, foodservice staff and patient representatives. The research team closely liaised with this group to co-develop the intervention, as well as research questions, methodologies, data collection approaches and implementation strategies. Lastly, the research team arranged regular face-to-face group and one-on-one meetings with staff on the study ward (in particular, colorectal surgical consultants) and the *Nutrition Reference Committee* to design the intervention over a 5-month period. The lead author acted as a knowledge broker by sharing existing evidence [1, 3–6, 33] and data generated from previous phases of the research [16–20] with knowledge users. The final intervention involved ten strategies aimed at organisational, HCP and patient levels, described briefly below and outlined in more detail in Table 1.

1. *Organisational level – introduction of a flexible EOF pathway:*
  - Free fluid diet (i.e. a diet inclusive of fluids and foods that are liquid at room temperature) prescribed and made available to the patient once deemed safe to consume liquids (~ 4 h after surgery);
  - One oral nutrition supplement (ONS) given upon patient return to the ward and deemed safe to consume liquids (~ 4 h after surgery);
  - High energy, high protein (HEHP) diet prescribed on postoperative day one (POD1) or thereafter, depending on the patient’s food preferences and clinical status; and
  - ONS prescribed three times a day (TDS) until discharge.
2. *Health Care Professional level – HCP awareness and education:*
  - Introduction to EOF pathway, including education sessions for nursing staff;
  - Introduction to EOF pathway, including education sessions delivered to interns, residents and registrars; and
  - Nutrition and diet orientation session delivered to interns and residents at the beginning of their rotation with the colorectal team.
3. *Patient level – engaging patients in their care:*
  - Delivery of verbal nutrition-related messages to patients during ward rounds;
  - Informing patients how to order meals on or after POD1 using the electronic foodservice system; and
  - Providing a one-page nutrition handout to patients pre-operatively (in surgical preadmission clinic), which outlined:
    i. The importance of nutrition for recovery from surgery;
    ii. Timing and types of drinks/foods to expect after surgery (e.g. liquids within 4 h after surgery); and
    iii. How to optimise nutrition intake in hospital.

**Table 1:** Intervention strategies

Strategy	Delivered by	Delivered to	Intended outcome	Additional information
**Organisational level (EOF pathway)**				
Free fluids prescribed on POD0	Doctor	Patient	Decrease times to first diet and solid diet prescription and improve energy and protein intakes.	EOF pathway was flexible and individualized, encouraging HCPs to exercise their clinical judgment and refer nutritionally ‘at risk’ patients to a dietitian. Prescription of ONS twice daily for the first three days after surgery was a strategy identified early on by knowledge users, who indicated it was acceptable and feasible. Due to conflicting perspectives from staff members, this strategy was removed from the formal EOF pathway, although surgeons adopted and implemented this strategy as they acknowledged the strength of the evidence supporting this practice.
Free fluids and ONS delivered POD0	Nurse	Patient		
HEHP diet prescribed POD1	Doctor	Patient		
ONS prescribed TDS	Doctor	Patient		
**Health care professional level**				
Awareness and education sessions	CF and lead author	Registered and enrolled nurses	Increase staff awareness around importance of timely and adequate nutrition after surgery; and familiarize staff with intervention strategies being implemented. Reduce times to first nutrition delivery and diet upgrades.^a^	Sessions delivered to nurses were formal (a 30–45 min PowerPoint presentation was utilized to share evidence for EOF and findings from previous study phases, six weeks prior to starting data collection) or informal (a 5–10 min overview of the information presented in the formal sessions was conducted during scrum meetings and ad hoc, at times convenient to staff, over 2-week period prior to data collection). Study flyers and an email containing information on the intervention and EOF pathway were disseminated to nursing staff one week prior to data collection.
Awareness and education sessions	Colorectal fellow	Interns, residents, registrars		
Ward orientation (nutrition)	Ward dietitian	Interns, residents		
**Patient level**				
Meal ordering awareness	Treating nurse or AIN	Patient	Inform patients about timely and adequate nutrition; and support them to participate in their nutrition care.	Delivery and content of nutrition-related messages were done at each HCP’s discretion. The handout was piloted among lay members of the public (*n* = 3) to assess readability, resulting in minor changes to wording, structure and design.
Nutrition-related messages	Doctor	Patient	Increase patient awareness of nutrition after surgery and encourage oral intake.	
Preoperative nutrition handout	Preadmission clinic nurse	Patient	Facilitate patient-centered care and increase oral intake by encouraging patients to select foods that they prefer/can manage.	

*AIN* Assistant in Nursing, *CF* Clinical Facilitator (nursing), *EOF* early oral feeding, *HEHP* high Energy, high protein, *ONS* oral nutrition supplement/s, *POD* postoperative day, *TDS* three times daily

^a^Nursing staff were responsible for sourcing foods on the ward; and medical staff were responsible for prescribing diets after surgery

### Sample

All consecutive patients who were: (i) able to provide written informed consent (aged ≥18 years, cognitively intact and able to communicate in English); and (ii) undergoing an elective colorectal and/or small bowel surgery, were approached by the lead author for inclusion in the study. Patients were excluded if they were critically ill (i.e. intubated, ventilated, admitted to/transferred from the intensive care unit). A convenience sample of 40–60 patients were planned to be recruited, based on the number of bowel resections anticipated to be performed over the 10 weeks allocated to collect data. Informed written consent was obtained from patients by the lead author in the surgical preadmission clinic approximately 1 week prior to surgery, or during the postoperative period.

A sample of HCPs were recruited to participate in interviews to explore their perceptions of the intervention. Any full- or part-time dietitian, nurse or doctor who provided care to patients on the ward during the period of the intervention were eligible to participate. With assistance from the clinical facilitiator, potential participants meeting the inclusion criteria were identified. Verbal informed consent was obtained from participating HCPs.

### Data collection

Descriptions of process evaluation domains and data collection methods are provided in Table 2. Quantitative data, including patient demographics and intervention reach and delivery were collected through chart reviews, verbal clarification and/or direct observations over a 10-week period (August to October 2018). The lead author independently collected all quantitative data to eliminate inter-rater variability. Qualitative data, including intervention fidelity (of nutrition-related messages) and HCPs responses to the intervention were collected between August to October 2018 and during December 2018, respectively. HCPs were selected for interviews using maximum variation purposive sampling to include a mix of ages, genders, professional roles and years of clinical experience. In interviews, HCPs were asked about their awareness of the study, and their perceptions around intervention acceptability and effects (Supplementary Material 4). A trained female research assistant with a background in nutrition, not known to staff, conducted interviews to minimize bias, as the lead author had contact with HCPs during intervention development. All staff were interviewed one-on-one, on-site at a time and place convenient to them. Interviews were digitally recorded (average: 22 min; range: 20–26 min) and transcribed verbatim for analysis.

**Table 2:** Process evaluation components and methods, adapted from Moore 2015

Evaluation domain		Description	Data collection methods
**Reach**	Recruitment of patients	Proportion of patients recruited of those eligible. (Q*uantitative data*)	Screening log: recorded all eligible patients approached and recruited during study period.
**Intervention delivery**	Delivery to patients	Quantity and quality of intervention delivery; i.e.: Were patients’ diets prescribed in accordance with EOF pathway? Did patients receive pre-operative nutrition handout and verbal nutrition-related messages? How long were the nutrition-related messages and what did they entail? (*Quantitative and qualitative data*)	Chart audits and direct observations to assess whether patients were prescribed diets in accordance with pathway, and if not, reasons why recorded. Chart audit determined whether preoperative nutrition handout was delivered. Direct observations^a^ assessed whether doctors provided nutrition-related messages and the content covered.
	Delivery to HCPs	Quantity and quality of intervention delivery; i.e.: How many staff attended sessions? What topics were covered? How long were sessions? (*Quantitative data*)	Intervention delivery log: recorded staff who attended information sessions, duration of each session, materials used, and content covered.
HCP responses and interactions		Staff responses to and interactions with intervention; mediators; and unexpected pathways and consequences. (*Qualitative data*)	Semi-structured interviews were undertaken by a research assistant at end of study period.
Context		Anything external to the intervention that may act as a barrier or facilitator to implementation or moderate its effects. (*Qualitative data*)	A description of the study site and participants was considered in the interpretation of findings.
Adaptations		Any tailoring, changes or adaptations made to the original intended intervention. (*Qualitative data*)	Field notes were kept by the lead author with details of any adaptations/ changes made to the original intervention.
Causal assumptions/ mechanisms		Data informing proposed causal assumptions/mechanisms sourced from process and outcome data (described within this table); as well as existing literature.	

*EOF* early oral feeding, *HCP* healthcare professional

^a^Via attending ward rounds every second week

### Data analysis

Qualitative and quantitative data were analysed simultaneously. All quantitative data were entered into SPSS Statistics for Windows version 23.0 (IBM Corp. 2012, Armonk, N.Y., USA). Continuous data were tested for normality (Shapiro-Wilk test). Data on intervention reach and delivery to both HCPs and patients were summarized using frequency and percent. Demographic data were summarized using median (interquartile range [IQR]) or mean ± standard deviation (SD) for continuous variables, and frequency/percent for categorical variables. Qualitative data were analyzed using inductive content analysis [34]. This involved the lead author (MR) reading and rereading the transcribed interviews and identifying codes from the data, which were grouped into subcategories, then categories. Data saturation was apparent after the seventh interview when no new information emerged. Analytic rigor and trustworthiness of findings were upheld by a) having regular discussions among the research team regarding emerging categories, for credibility of findings; and b) maintaining memos throughout data analysis to document analytical decisions made, for dependability [35].

## Results

### Reach

Overall, 40 patients were recruited from a total of 42 approached (95%). Six patients were excluded from the study due to either having their operation postponed/cancelled (*n* = 5) or being transferred to the ICU (*n* = 1). Thus, complete data were collected for 34 (85%) recruited patients. The majority of participants were male (*n* = 22, 65%), had an anastomosis formed (*n* = 24, 71%) and underwent a laparoscopic/robotic surgery (*n* = 27, 79%). Participants’ mean age was 60.6 ± 13.3 years and the most common surgeries performed were high anterior resection (*n* = 10, 29%), right hemicolectomy (*n* = 9, 26%) and ultra-low anterior resection (*n* = 4, 12%).

### Intervention delivery

Variance in delivery of the ten intervention strategies was observed (Table 3), particularly with strategies targeted at HCPs. All patients received at least one of the seven strategies targeted at organisational and patient levels.

**Table 3:** Intervention delivery among staff and patients

Intervention strategies	Number (%) who received	Reasons for non-compliance
Organisational level (EOF pathway)		
Free fluids diet prescribed on POD0	27 (79%) patients	•Clear fluid diets were used due to extensive adhesiolysis (n = 3); and no reason given/observed (n = 4).
ONS delivered POD0	21 (62%) patients	•Not prescribed (n = 5); patient admitted to ward following ONS prescription cut-off time (*n* = 2); patient nil-by-mouth (n = 1); unsuitable ONS formula for dietary requirements (*n* = 1); and no reason given/observed (n = 4).
HEHP diet prescribed POD1	0 (0%) patients	•No explanation provided in patients’ medical records. However, interview data revelled staffs’ views on prescribing solids after surgery differed greatly from this.
ONS prescribed TDS^a^	34 (100%) patients	Not applicable.
HCP level (education/training)		
Awareness and education session(s)	41 (84%) nurses	•Missed session due to shift rotation (*n* = 8).
Awareness and education session(s)	0 (0%) doctors	•Unclear, however, it was noted the colorectal fellow went on unanticipated leave for approximately one week at the start of data collection.
Ward orientation (nutrition) session(s)	0 (0%) doctors	•Unclear, however, the staff members mentioned in Table 1 who had conflicting views of the intervention were managerial level members of dietetics which may have influenced intervention delivery by the ward dietitian.
Patient level (engagement in care)		
Meal ordering awareness	17 (50%) patients	•No reason recorded, however, likely attributed to unclear responsibilities (e.g. one week prior to data collection, this role became a shared responsibility between assistant in nursing staff and each patients’ treating nurse).
Nutrition-related messages	23^b^ (92%) patients	•No reason observed (*n* = 2).
Preoperative nutrition handout	25 (74%) patients	•Appointment occurred off-site (*n* = 1) or over the phone (n = 1); and no reason documented (*n* = 7).

*EOF* early oral feeding, *HEHP* high energy, high protein, *HCP* health care professional, *ONS* oral nutrition supplements, *POD* postoperative day, *TDS* three times a day

^a^Strategy adopted by staff

^b^Subset of patients included in study (*n* = 25)

#### Delivery among HCPs

##### Awareness and education sessions

Overall, 41 (84%) registered and enrolled nursing staff received an awareness and education session; 21 (43%) and 20 (41%) nurses received a formal and informal session, respectively. No formal awareness and educations sessions were held with interns, residents or registrars.

##### Ward orientation session

Formal nutrition orientation sessions were not held with interns or residents during the time allocated to collect data.

#### Delivery among patients

##### Free fluids from POD0

Over three-quarters of the cohort (*n* = 27) were prescribed free fluids on POD0. When free fluids were not prescribed on POD0, clear fluids were used among six patients, while one patient was nil-by-mouth. Of the 27 patients prescribed free fluids, 15 (56%) were delivered free fluid items (excluding ONS) on POD0. Reasons why patients did not receive free fluids on POD0 included: nausea (*n* = 1); late admission to ward and patient too drowsy (*n* = 4); and no reason recorded (*n* = 7).

##### ONS delivered on POD0

Twenty-one patients (62%) were delivered one ONS on POD0.

##### HEHP diet prescribed on POD1

No patients were prescribed a HEHP diet on POD1. One patient was prescribed a soft diet on POD1 after requesting food. Three-quarters of the cohort were prescribed a HEHP at some point during their stay, ranging from POD2–POD14.

##### ONS prescribed TDS

All patients were prescribed ONS TDS during their hospital stay. Most patients (*n* = 24, 71%) were prescribed ONS on POD0 (for commencement on POD1). The remaining patients were prescribed ONS on POD1 (*n* = 9, 26%) and POD2 (*n* = 1, 3%).

##### Meal ordering awareness

In total, 22 (65% of) patients were aware of how to order their meals via the electronic foodservice system by discharge. Most (*n* = 17, 50%) were informed by staff, while five patients were aware of how to order meals from a previous admission or were self-taught.

##### Pre-operative nutrition handout

The one-page nutrition handout was given to 25 (74%) patients before their surgery.

##### Nutrition-related messages

At least one ward round, in which the surgical team verbally delivered care, was observed per patient for 25 (74%) enrolled patients. Of these patients, 23 (92%) received a nutrition-related message from medical staff during their stay. The two most common messages noted involved encouraging ONS consumption and emphasising slow and cautious introduction of foods and fluids. These messages were often short (~ 10–15 s) and generally delivered by a registrar or fellow on POD1 or POD2 when patients were on a free fluid diet.

### Staffs’ responses to the intervention

Nine HCPs participated in interviews, including five nurses (three registered nurses, one team leader and one enrolled nurse), three doctors (two registrars and one fellow) and one dietitian. Every staff member approached agreed to participate. Staffs’ perceptions of the intervention’s acceptability and impact are described in Table 4. Staff also provided insight into how the intervention could be improved and sustained in usual practice, such as: greater education and awareness of nutrition and the EOF pathway among doctors; continuing in-services among nursing staff; reducing complexity/wording of EOF pathway; formal implementation of adjunct ERAS components; and offering alternative ONS or drinks (e.g. flavoured milk) to patients who disliked the commercial ONS.

**Table 4:** Staffs’ responses to intervention components

**Perceptions of the intervention**	
(1) Nutrition-related messages are important, but require improvement	
While staff from all disciplines spoke about the importance of doctors providing nutrition-related messages to patients, there was consensus that the specificity of these messages could be improved. Some staff suggested dietitians should provide this specific advice, considering doctors receive minimal training around nutrition.	
“*I think it’s good that [doctors] are able to provide that information to the patients, but there’s still that confusion from patients about ‘what can I have when I go home’ and ‘what can I have when I’m here?’ … but I think it’s good that they’re at least having some involvement with [patients’] nutrition.*” (P02, Nurse, F).	
(2) Information sessions were successful at increasing awareness among nurses	
Staff said the information sessions were successful in achieving widespread awareness of the intervention among nurses. This in turn appeared to increase nurses’ acceptance of the strategies implemented, with staff highlighting positive changes in ward practices and attitudes towards postoperative nutrition delivery.	
“*I think we had a pretty good work up to it … and I think most of the staff were pretty well aware of it … [The purpose of the study was] to protect them [patients] from nutrition deficiencies immediately post-op and rehabilitate their gut quicker.*” (P04, Nurse, F).	
(3) Dietary prescription preferences differ to those outlined in the pathway	
Doctors, particularly registrars, spoke of ideally prescribing solids after patients had passed wind/and or opened their bowels, and using a soft diet prior to prescribing a full diet, to test patients’ gut tolerance; criteria which were not outlined in the EOF pathway.	
“*I think there is healthy medical belief, maybe not held in any great evidence-base, but a healthy medical belief that until the bowels are opening, until something is coming out of the back end, at least wind, and we know that the bowels are working, putting things [food and liquid] in the top end is kind of stupid.*” (P08, Doctor, M).	
(4) Divergent views on the generic prescription of ONS	
Staffs’ responses to the blanket prescription of ONS among elective colorectal patients ranged from high to minimal support. Some staff held the belief that ONS should only be used where indicated and food should be encouraged first.	
“*I definitely think we should be encouraging patients with food first … So, I would be going with food first, then supplements.*” (P06, Dietitian, F). “*I think it’s good that the option is there, and whether or not the patient can tolerate it or not, can deal with that when it comes.*” (P02, Nurse, F).	
**Perceived impact of the intervention**	
(1) Improved initial nutrition prescription, but minimal change in diet progression	
Staff described how the intervention facilitated improved nutrition prescription (e.g. greater use of ONS and HEHP diets) early in patients’ admissions, however, minimal change was acknowledged in regard to progressing patients through the different postoperative diets (e.g. from liquids to solids). Additionally, the widespread prescription of ONS appeared to be driven by an influential senior doctor on the ward.	
“*I think I did become more aware of making sure we did understand...that we need to put patients on high protein diets.*” (P09, Doctor, M). “*The only change I have seen … has come from a person who … in the vast majority of cases is the one guiding ward rounds and in control of bedside patient care … [who] began demanding that all of [their] juniors chart Resource [an ONS] on a regular basis.*” (P08, Doctor, M).	
(2) Greater awareness of and responsibility for nutrition among HCPs	
Many participants, particularly those present on the ward pre-intervention, perceived that the intervention had made staff more conscious of nutrition. Further, it appeared that nurses had increased interest in confirming/checking patients’ diet prescriptions with doctors.	
“*Overall, I think the ward is more aware that people should be eating; even the nurses sometimes will remind us, you know, ‘can you upgrade their diet?’, rather than just leaving everything to us.*” (P09, Doctor, M).	
(3) Enhanced patient participation in care	
Some staff associated the intervention with improving patients’ psychological wellbeing (due to nutrition being available earlier) and facilitating patient participation in care (a result of patients being more aware of nutrition).	
“*I think it’s probably better for the patient’s wellbeing, and like I said one of the first things they like to ask you is ‘when can I eat?’ So, I think being able to give them something that’s a bit substantial is good for them, rather than just some jelly, you know. That really helps them mentally.*” (P03, Nurse, F).	

*EOF* early oral feeding, *ERAS* Enhanced Recovery After Surgery, *F* female, *M* male, *ONS* oral nutrition supplements

## Discussion

This study described the co-development and process evaluation of a complex intervention, designed with knowledge users, to improve oral intake among patients who undergo elective bowel surgery. Recruitment of patients was broad, providing representation of different age, sex and surgical procedure types. Delivery of intervention components varied considerably among patients (0–100%) and HCPs (0–84%). Differences in HCPs’ involvement in designing and awareness of the intervention appeared to influence how it was implemented and accepted in practice. These findings provide useful insights for HCPs and researchers seeking to implement evidence-based nutrition guidelines in their own settings.

This paper highlights the utility of an iKT approach to design and implement complex interventions for translating evidence into clinical practice. It also provides learnings on how this might be best achieved, which may be useful to others considering there is limited evidence about how researchers and knowledge users should go about collaborating. The combination of engaging knowledge users through establishing a multidisciplinary *Nutrition Reference Committee* and having regular, in-person discussions with staff on the study ward likely explain why certain patient-related strategies were delivered well. For example, a fellow, who held influence on the ward and was involved in regular face-to-face meetings regarding intervention design, was an advocate for the prescription of ONS and the delivery of nutrition-related messages on the ward; strategies which were delivered to ≥92% of patients. Further, the prescription of free fluids on POD0 was widely adopted by staff, which is likely attributed to the consultants, who contributed to intervention design and who were present in theatre where first diet types are prescribed. Similarly, previous work has reported high adherence rates to nutrition-related intervention components in habitual practice when knowledge users are engaged in development, implementation and evaluation [36–38]. These may be attributed to knowledge users taking ownership of the end product, from being part of the design process; and their input likely resulted in the development of strategies they perceived to be effective and acceptable [21]. Further, the collaborative approach fostered by the research team within the current project likely facilitated the adoption of aforementioned strategies. For example, during Phase 3, the research team ensured that messages were tailored to the specific target audience, clear and concise tools were developed to enable HCPs to easily interpret previous research findings (e.g. one-page summary handouts) and regular in-person contact was made with knowledge users; factors which have been identified as enablers of iKT [39]. In fact, in-person contact with researchers has been widely acknowledged by knowledge users as the most influential factor determining their use of research evidence [40]. Therefore, these factors likely explain why certain patient-related interventions were well delivered by doctors and nurses.

However, not all strategies were well implemented. The variable delivery of awareness and education sessions may help explain the mixed uptake of some of the patient-related strategies, and HCPs’ responses to the intervention. For example, many nurses were involved in refining intervention strategies, and most received an awareness and education session (84%), which can be attributed to the nursing clinical facilitator who was a member of the *Nutrition Reference Committee* and delivered these sessions with assistance from the lead author. This appeared to directly increase nurses’ acceptability of, and support for the intervention, as demonstrated in their responses and in observational data, where 78% of patients were delivered free fluids on POD0. Alternatively, all registrars, residents and interns were new to the ward and thus were not involved in designing the intervention. Further, these staff did not receive any awareness and education or ward orientation sessions. It was noted that the colorectal fellow, who was responsible for delivering the education sessions went on unanticipated leave during the first week of data collection and her reasonability was not backfilled, while consensus about the intervention had not been achieved within the dietetics department at the time of implementation, which may have influenced the ward dietitians’ intention to deliver the ward orientation sessions. In part, this may explain why no enrolled patients were prescribed solids on POD1, as outlined in the EOF pathway. While this strategy may not be appropriate for all patients [1–3] or in line with their preferences [18], it was evident from HCPs’ responses and observational data that registrars’ views on prescribing solids after surgery differed greatly from what was outlined in the EOF pathway and supported by EBGs. While previous work has found educational efforts successful in facilitating change [41], it is unclear whether our awareness and education sessions, intended to introduce the pathway and provide evidence for EOF, would have been sufficient to change the behavior of medical staff. It is possible that further behavioral strategies may have also been required given the complex nature of human behavior in clinical environments (e.g. social influence/beliefs about consequences/knowledge/memory, attention and decision processes) [42]. However, it does demonstrate that while it is important to gain buy-in from influential knowledge users early in the planning process to facilitate change, involving all clinical staff who are involved in prescribing and delivering nutrition care was required for acceptance, and thus widespread adoption of the intervention, which was not demonstrated in registrars’ or dietitians’ responses.

Three patient-related intervention strategies, including providing ONS on POD0, information around meal ordering on POD1 or thereafter, and a nutrition handout prior to surgery were delivered to 50–74% of patients. Anecdotally it was observed that system barriers and unclear responsibilities were the reasons behind why these strategies were not implemented with greater success. For example, assistant in nursing staff were originally appointed the role of informing patients how to order their meals; however, one week prior to data collection, this role became a shared responsibility between assistant in nursing staff and each patients’ treating nurse. Indeed, previous work has found that misaligned goals, roles and responsibilities are barriers to iKT [29]. Therefore, future work should ensure responsibilities are clearly outlined and held accountable, and risk-management plans are in place for managing system errors.

While findings generated from this study provide important insights into the development and implementation of a multifaceted nutrition intervention for translating evidence into usual care, it has some limitations. For example, the evaluator was initially involved in facilitating the design and implementation of intervention strategies, and therefore their presence on the ward may have impacted HCPs’ behaviours during evaluation. Future work should consider employing research assistants (who are not involved in intervention design or implementation) to collect data to minimise bias. This was not possible in the current study due to resource constraints. Further, this study was conducted on one ward, therefore the findings may not be generalizable given contextual factors. Lastly, while a summary of this intervention’s effects are provided in detail elsewhere [43], further refinements and testing are required to determine whether the intervention is clinically and cost effective before widespread implementation. For example, incorporating strategies to support clinicians in enacting a more person-centered approach to postoperative nutrition care should be considered in future work, given this has previously been identified as an important factor by patients [18, 43].

## Conclusion

This study described the co-development and process evaluation of a complex intervention designed to improve the timey and adequacy of nutritional intakes among patients following bowel surgery. The multifaceted intervention, which was co-developed with knowledge users at the study hospital over a 5-month period, included ten strategies, targeted at staff or patients. The process evaluation revealed that certain patient-related strategies were well adopted by HCPs (e.g. nutrition related messages and ONS prescribed TDS), while others were not (e.g. HEHP diet prescribed POD1 and meal ordering awareness). Further, staffs’ awareness of and acceptance toward the intervention varied greatly. This appeared to be due to variability in the delivery of staff-related strategies (e.g. awareness and education sessions), in conjunction with staffs’ direct involvement in co-designing the intervention. These findings suggest future work intending to use an iKT approach to design and implement clinical interventions should place high importance on end-user engagement in order to optimise intervention success. This work provides useful insights for HCPs and/or researchers seeking to implement nutrition guidelines in their own settings.

## Supplementary Information

**Additional file 1.**

**Additional file 2.**

**Additional file 3.**

**Additional file 4.**

## Data Availability

The datasets during and/or analysed during the current study available from the corresponding author on reasonable request.

## Abbreviations

EBGs: Evidence-based guidelines
EOF: Early oral feeding
HCPs: Healthcare professionals
HEHP: High energy, high protein
iKT: Integrated knowledge translation
KTA: Knowledge to Action
KT: Knowledge translation
ONS: Oral nutrition supplements
PEM: Protein-energy malnutrition
POD: Postoperative day
SD: Standard deviation
TDS: Three times a day
IQR: Interquartile range
