# Supplementary material for: Assessment of an integrated knowledge translation intervention to improve nutrition intakes among patients undergoing elective bowel surgery: a mixed-method process evaluation
Source: BMC Health Serv Res. 2021 May 27;21:514. doi: 10.1186/s12913-021-06493-2 (PMC8161936; doi:10.1186/s12913-021-06493-2)
Supplement: Supplementary file 1 — Additional file 1. [file 12913_2021_6493_MOESM1_ESM.docx]

**Supplementary Material 1: The TIDieR (Template for Intervention Description and Replication) Checklist**

| **Item number** | **Item** | **Where located **** | |
| --- | --- | --- | --- |
|  |  | Primary paper  (page or appendix  number) | Other ^†^ (details) |
|  | **BRIEF NAME** |  |  |
| **1.** | Provide the name or a phrase that describes the intervention. | 1 |  |
|  | **WHY** |  |  |
| **2.** | Describe any rationale, theory, or goal of the elements essential to the intervention. | 5-7 and Table 1 |  |
|  | **WHAT** |  |  |
| **3.** | Materials: Describe any physical or informational materials used in the intervention, including those provided to participants or used in intervention delivery or in training of intervention providers. | 5-7 and Table 1 |  |
| **4.** | Procedures: Describe each of the procedures, activities, and/or processes used in the intervention, including any enabling or support activities. | 5-7 and Table 1 |  |
|  | **WHO PROVIDED** |  |  |
| **5.** | For each category of intervention provider (e.g. psychologist, nursing assistant), describe their expertise, background and any specific training given. | Table 1 |  |
|  | **HOW** |  |  |
| **6.** | Describe the modes of delivery (e.g. face-to-face or by some other mechanism, such as internet or telephone) of the intervention and whether it was provided individually or in a group. | Table 1 |  |
|  | **WHERE** |  |  |
| **7.** | Describe the type(s) of location(s) where the intervention occurred, including any necessary infrastructure or relevant features. | Page 4 |  |
|  | **WHEN and HOW MUCH** |  |  |
| **8.** | Describe the number of times the intervention was delivered and over what period of time including the number of sessions, their schedule, and their duration, intensity or dose. | Table 1 and Table 2 and pages 8-11 |  |
|  | **TAILORING** |  |  |
| **9.** | If the intervention was planned to be personalised, titrated or adapted, then describe what, why, when, and how. | Table 1 |  |
|  | **MODIFICATIONS** |  |  |
| **10.** | If the intervention was modified during the course of the study, describe the changes (what, why, when, and how). | Table 1 |  |
|  | **HOW WELL** |  |  |
| **11.** | Planned: If intervention adherence or fidelity was assessed, describe how and by whom, and if any strategies were used to maintain or improve fidelity, describe them. | Page 7 and Additional file 3 |  |
| **12.** | Actual: If intervention adherence or fidelity was assessed, describe the extent to which the intervention was delivered as planned. | Table 2 and pages 8-11 |  |
