# Supplementary material for: Assessment of an integrated knowledge translation intervention to improve nutrition intakes among patients undergoing elective bowel surgery: a mixed-method process evaluation
Source: BMC Health Serv Res. 2021 May 27;21:514. doi: 10.1186/s12913-021-06493-2 (PMC8161936; doi:10.1186/s12913-021-06493-2)
Supplement: Supplementary file 2 — Additional file 2. [file 12913_2021_6493_MOESM2_ESM.docx]

| **Supplementary Material 2: Consolidated criteria for reporting qualitative studies (****COREQ)** | | | | |
| --- | --- | --- | --- | --- |
| No | Item | Guide questions/descriptions | Page no | Comment |
| **Domain 1: Research team and reflexivity** | | | | |
| **Personal Characteristics** |  |  |  |  |
| 1. | Interviewer/facilitator | Which author/s conducted the interview or focus group? | 9 | A trained research assistant conducted the interviews |
| 2. | Credentials | What were the researcher’s credentials? E.g. PhD, MD | 9 | The research assistant had a nutrition background and was trained in interviewing techniques |
| 3. | Occupation | What was their occupation at the time of the study? | 9 | Research assistant |
| 4. | Gender | Was the researcher male or female? | 9 | Female |
| 5. | Experience and training | What experience or training did the researcher have? |  | The authors were doctorally prepared health researchers trained in qualitative techniques, while the research assistant had experience with interviewing |
| **Relationship with participants** |  |  |  |  |
| 6. | Established relationship | Was a relationship established prior to study commencement? | 9 | The research assistant was not known to staff |
| 7. | Participant knowledge of the interviewer | What did the participants know about the researcher? e.g. personal goals, reasons for doing the research? | 7 | All participants were informed about the aim of this study and that they had the right to withdraw at any time |
| 8. | Interviewer characteristics | What characteristics were reported about the interviewer/facilitator? e.g. Bias, assumptions | 9 | She was not known to staff therefore minimising bias |
| **Domain 2: study design** | | | | |
| **Theoretical framework** |  |  |  |  |
| 9. | Methodological orientation and theory | What methodological orientation was stated to underpin the study? e.g. grounded theory, discourse analysis, ethnography, phenomenology, content analysis | 9 | Content analysis |
| **Participant selection** |  |  |  |  |
| 10. | Sampling | How were participants selected? e.g. purposive, convenience, consecutive, snowball | 9 | Maximum variation purposive sampling was used to include a mix of ages, genders, professional roles and years of clinical experience |
| 11. | Method of approach | How were participants approached? e.g. face-to-face, telephone, mail, email | 7 | With assistance from the clinical nurse educator, potential participants meeting the inclusion criteria were identified |
| 12. | Sample size | How many participants were in the study? | 13 | Nine |
| 13. | Non-participation | How many people refused to participate or dropped out? Reasons? | 13 | Every staff member approached agreed to participate |
| **Setting** |  |  |  |  |
| 14. | Setting of data collection | Where was the data collected? e.g. home, clinic, workplace | 9 | All staff were interviewed one-on-one, on-site at a time and place convenient to them. |
| 15. | Presence of non-participants | Was anyone else present besides the participants and researchers? | 9 | All staff were interviewed one-on-one, on-site at a time and place convenient to them. |
| 16. | Description of sample | What are the important characteristics of the sample? e.g. demographic data, date | 13 | Of the staff interviewed, five were nurses (three registered nurses, one team leader and one enrolled nurse), three were doctors (two registrars and one fellow) and one was a dietitian. |
| **Data collecting** |  |  |  |  |
| 17. | Interview guide | Were questions, prompts, guides provided by the authors? Was it pilot tested? | 9 | Yes – Supplementary material 5 |
| 18. | Repeat interviews | Were repeat interviews carried out? If yes, how many? | - | No |
| 19. | Audio/visual recording | Did the research use audio or visual recording to collect the data? | 9 | Interviews were digitally recorded (average: 22 minutes) and transcribed verbatim for analysis. |
| 20. | Field notes | Were field notes made during and/or after the interview or focus group? | 10 | Memos were maintained throughout data analysis to document analytical decisions made using direct quotes from participants to support findings |
| 21. | Duration | What was the duration of the interviews or focus group? | 9 | Interviews were digitally recorded (average: 22 minutes) and transcribed verbatim for analysis. |
| 22. | Data saturation | Was data saturation discussed? | 10 | Data saturation was apparent after the seventh interview when no new information emerged |
| 23. | Transcripts returned | Were transcripts returned to participants for comment and/or correction? | - | No |
| **Domain 3: analysis and findings** | | | | |
| **Data analysis** |  |  |  |  |
| 24. | Number or data coders | How many data coders coded the data? | 9 | One (MR) |
| 25. | Description of coding tree | Did authors provide a description of the coding tree? |  | Transcripts were analysed using Braun and Clark’s six-step guide to thematic analysis (50): (i) familiarisation with the data; (ii) generating initial codes; (iii) searching for themes; (iv) reviewing themes; (v) defining and naming themes and; (vi) writing the report |
| 26. | Derivation of themes | Were themes identified in advance or derived from the data? | 9 | Derived from the data – inductive content analysis was used. |
| 27. | Software | What software, if applicable, was used to manage the data? | 9 | SPSS for numerical demographic data |
| 28. | Participant checking | Did participants provide feedback on the findings? | - | No |
| **Reporting** |  |  |  |  |
| 29. | Quotations presented | Were participant quotations presented to illustrate the themes / findings?  Was each quotation identified? e.g. participant number | Yes | Table 3 |
| 30. | Data and findings consistent | Was there consistency between the data presented and the findings? | Yes |  |
| 31. | Clarity of major themes | Were major themes clearly presented in the findings? | Yes | Table 3 |
| 32. | Clarity of minor themes | Is there a description of diverse cases or discussion of minor themes? | Yes | Page 13 |
