# Supplementary material for: Assessment of an integrated knowledge translation intervention to improve nutrition intakes among patients undergoing elective bowel surgery: a mixed-method process evaluation
Source: BMC Health Serv Res. 2021 May 27;21:514. doi: 10.1186/s12913-021-06493-2 (PMC8161936; doi:10.1186/s12913-021-06493-2)
Supplement: Supplementary file 3 — Additional file 3. [file 12913_2021_6493_MOESM3_ESM.docx]

| **Supplementary Material 3. Researchers and knowledge users involved in intervention development** | | | | |
| --- | --- | --- | --- | --- |
|  | **Researchers** | **Knowledge users** | | **Engagement** |
|  |  | Clinicians | Patients |  |
| Study team | Professor of Nursing  Research Fellow (N&D)  A/Prof of N&D  PhD Candidate (N&D) | Director of General Surgery (study ward) | N/A | Involved in all aspects of research (study design and intervention development, implementation, evaluation) |
| *Nutrition Reference Committee* | As above | Director of Trauma  Manager of Foodservices  Director of N&D  Clinical facilitator (study ward)  Nurse Unit Manager (study ward)ᵃ  2 x senior clinical dietitians | 2 x previous surgical patients (orthopaedic and colorectal) | Co-created research questions, data collection approaches and intervention strategies |
| Staff on study ward | N/A | 1 x registrarᵃ  1 x colorectal fellow  5 x colorectal consultants  Ward dietitian  Enrolled and registered nursing staff | N/A | Co-created intervention strategies |
| N/A, Not applicable; N&D, Nutrition and Dietetics.  ^a^Rotated or left the ward prior to process evaluation. | | | | |
