# Supplementary material for: Assessment of an integrated knowledge translation intervention to improve nutrition intakes among patients undergoing elective bowel surgery: a mixed-method process evaluation
Source: BMC Health Serv Res. 2021 May 27;21:514. doi: 10.1186/s12913-021-06493-2 (PMC8161936; doi:10.1186/s12913-021-06493-2)
Supplement: Supplementary file 4 — Additional file 4. [file 12913_2021_6493_MOESM4_ESM.docx]

| **Supplementary Material 4: Semi-structured interview guide** |
| --- |
| Awareness of the feeding intervention study |
| Were you aware that a nutrition intervention study was happening among postoperative colorectal patients?  From your understanding, what was the purpose of the nutrition study/intervention?  If any, what were the main messages you took from the nutrition study/intervention?  How aware do you think your peers and other disciplines were of the nutrition intervention/study? |
| Perceptions of the feeding intervention |
| Overall, what are your thoughts on the nutrition study/intervention?  What was good or bad about the nutrition study/intervention?  How successful or unsuccessful do you think the nutrition study/intervention was?  Were there intervention components that worked better or were more useful than others?  - What did you think about the nutrition pathway?  - What did you think about doctors factoring in patients’ food preferences when determining if they are   ready to have solids prescribed (e.g. soft, full, high protein high energy)?  - What did you think about patients being prescribed a high protein diet?  - What did you think about doctors providing nutrition-related messages to patients?  - What did you think about all patients being prescribed oral nutrition supplements after surgery?  - What did you think about the information sessions about feeding after surgery? |
| Perceived impact of the feeding intervention |
| Overall, what was the impact of the nutrition study/intervention on this ward?  If any, what were the benefits or limitations of this ward being involved in this study/intervention?  How were staff affected by the nutrition study/intervention? |
| Utility of feeding intervention in continued practice |
| Why should this intervention continue or discontinue on this ward?  If any, what barriers or enablers do you see for continued use of the intervention in daily practice?  How can the intervention be sustained on this ward?  What further changes need to be made to improve nutrition after surgery for patients? |
| **Note**: Generic prompts include: “Can you tell me more about this?”; “Can you explain this further / expand on this?” and “What do you mean when you say ____?” |
